# Nursing students’ appreciation of clinical supervision during work-integrated learning

**DOI:** 10.4102/hsag.v30i0.2985

**Published:** 2025-06-09

**Authors:** Mphambanyeni J. Mugwari, Neltjie C. van Wyk, Ndivhaleni R. Lavhelani

**Affiliations:** 1 Department of Nursing Science, Faculty of Health Sciences, University of Pretoria, Pretoria, South Africa

**Keywords:** appreciative inquiry, clinical supervision, nursing students, work-integrated learning, nursing education, theory-practice integration

## Abstract

**Background:**

Effective clinical supervision in learning-conducive environments is required to enhance students’ work-integrated learning. Conversely, ineffective clinical supervision can jeopardise students’ learning and skills development.

**Aim:**

The aim of the study was to explore and describe the aspects of clinical supervision during work-integrated learning that students of a designated nursing education institution appreciated.

**Setting:**

The study was conducted at an institution in South Africa that offered a 3-year diploma programme in nursing science. The students took part in work-integrated learning at five public hospitals and 20 clinics.

**Methods:**

Descriptive qualitative research with an appreciative inquiry approach was done. Second and third-year students were purposively selected and took part in four focus group discussions. Data saturation determined the sample size of 45 participants. The 5-D Appreciative Inquiry Model applied.

**Results:**

The participants appreciated opportunities to learn how to integrate theory in practice under the supervision of professional nurses in learning-conducive clinical environments and enabling relationships with facilitators and lecturers. They wished for ongoing cooperation between the institutions involved to ensure well-resourced facilities and manuals for standardised nursing procedures.

**Conclusion:**

Nursing students require clinical supervision to benefit optimally from work-integrated learning. The supervision should be provided by hospital and clinic staff, as well as clinical facilitators and lecturers in learning-conducive environments created through cooperation between the institutions involved in the students’ training.

**Contribution:**

The research findings indicate the support that students require to use learning opportunities to become clinically skilled in integrating theory and practice.

## Introduction

Work-integrated learning aims to create opportunities for students to acquire nursing knowledge, develop clinical skills and learn how to interact with patients (González-García et al. 2021:695). Being students who learn to become professional nurses, they require clinical supervision from professional nurses during work-integrated learning (Achempim-Ansong, Kwashie & Ofei 2022:15; Snowdon et al. 2019:7).

Effective clinical supervision rests upon two pillars. The first pillar refers to the support that students get from professional nurses, including clinical facilitators and lecturers, and the second pillar refers to the collaborative engagement of the nursing education institution (NEI) and the healthcare facility where the students are engaged in work-integrated learning (Reynolds & Mclean 2023:2). The first pillar depends on positive professional nurse–student relationships (Kumudumalee et al. 2021:1; Port 2019:181) and the commitment from professional nurses to support students (Musabyimana et al. 2019:199). They require safe spaces to take part in patient care (Anggeria & Damanik 2022:525; Coleman & Hyde 2022:996) under the supervision of professional nurses who facilitate their development through constructive feedback (Bwanga & Mwansa 2022:644). Students depend on professional nurses to protect them from intimidation by others during work-integrated learning (Vizcaya-Moreno & Perez-Canaveras 2020:8) and appreciate professional nurses who adjust supervision according to individual students’ unique learning needs (Kelly & Hassett 2021:8; Keshavarzi 2022:05).

The second pillar depends on the institutions’ endeavours to cooperate in the planning and execution of students’ work-integrated learning and supervision during learning opportunities. In this regard, Fook (2016:2) is of the view that optimal clinical supervision provides students with opportunities to voice out their concerns about inadequate or inefficient patient care. Experienced professional nurses should be employed to manage clinical learning opportunities (Bwanga & Chanda 2019:135; Lee & Kim 2020:11) and create positive learning environments (Sithole 2017:22).

In the absence of optimal clinical supervision, students’ work-integrated learning is compromised to the extent that they may find it challenging to gain knowledge and become clinically skilled (Motsaanaka, Makhene & Ally 2020:5). The nursing students at the designated nursing education institution often complained about the clinical supervision challenges that they experienced during work-integrated learning. Because of a paucity of literature regarding what nursing students appreciate in clinical supervision existed and the first author supported by her research supervisors (the second and third authors) decided to involve nursing students to an appreciative inquiry about ideal clinical supervision during work-integrated learning. The aim of this study, therefore, was to explore and describe the aspects of clinical supervision during work-integrated learning that nursing students of a designated nursing education institution appreciated.

### Theoretical framework

**FIGURE 1:**
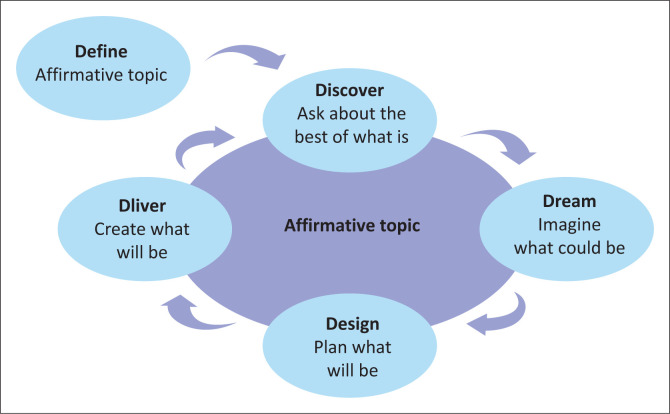
The 5-D appreciative inquiry model.

The 5-D appreciative inquiry model (Figure 1), developed by Cooperrider and Srivasta (1987), was used as a framework to guide the data collection and analysis processes. The model focusses on the appreciation of the strengths of institutions and their functioning such as clinical supervision during work-integrated learning instead of identifying deficits. Armstrong, Holmes and Henning (2020:1) considered appreciative inquiry as a valid way in assisting participants to move from a deficit-based process to a strength-based perspective. The model consists of five steps referring in this study to defining clinical supervision during work-integrated learning, discovering existing positive attributes, dreaming about the ideal process, designing such a process and describing means to deliver it.

## Research methods and design

An exploratory, descriptive, qualitative design using an appreciative inquiry framework was employed (Mugwari 2024:11). The constructivist paradigm applied assumes a relativist ontology (multiple realities), a subjectivist epistemology (knower and respondent co-create understandings) and a naturalistic (in natural world) set of methodological procedures (Denzin & Lincoln 2003:35). The researchers believed that nursing students hold different perspectives of clinical supervision in work-integrated learning and that, through interaction with the first author, they would share these perspectives while participating in work-integrated learning.

### Research setting

The study was conducted at a designated nursing education institution in the Gauteng province of South Africa that offered a 3-year diploma programme in nursing science. At the time of the study, 11 clinical supervisors and 11 theory lecturers assisted students with their training to become professional nurses. The students took part in work-integrated learning at five public hospitals and 20 clinics.

### Study population and sampling strategy

From the study population comprising 145 level two and three students, 45 participants were purposively selected. All the selected participants were capable to provide rich data about students’ appreciation of clinical supervision during work-integrated learning. Inclusion criteria required that participants be level two and three students regardless of age or gender, have had experience in clinical supervision and being able to comprehensively describe their perspectives about the studied phenomenon. Potential participants were invited and requested to consent voluntarily to participate in the study (Mugwari 2024:11).

### Data collection

Our focus group discussions, two with students from level two and two with students from level three, were done at times and venues agreed with the participants. Each group comprised 9 to 12 students, and the discussions, lasting about 120 min, were facilitated by the first author, supported by a co-facilitator who was also responsible for writing field notes. The following questions guided the discussions:

- *What does clinical supervision mean to you*? (definition phase of appreciative inquiry).
- *What are the best clinical supervision experiences you can appreciate*? (discovery phase of appreciative inquiry).
- *What are your wishes for an ideal clinical supervision*? (dream phase of appreciative inquiry).
- *How ideal clinical supervision could be achieved*? (design phase of appreciative inquiry).
- *How best do you think clinical supervision can be implemented in future*? (destiny phase of appreciative inquiry).

The discussions were audio-recorded with the permission of the participants and later transcribed verbatim by the first author. The field notes captured data that were not audio-recorded and helped compensate for any discrepancies. Data saturation was reached after four focus group discussions involving 45 participants.

### Data analysis

A six-step analysis designed by Cardiff, McCormack and McCance (2018:510) was applied. In the step called ‘familiarisation and submergence’, the transcripts and fieldnotes were read and re-read to gain an understanding of the data. During the second step that refers to ‘creative understanding’, the first author worked intermittently through the data to ensure that she returned with ‘new eyes’ to the data to identify keywords and concepts to indicate the information that was eventually used in the final set of categories and sub-categories. During ‘blending and melding’, the keywords and concepts were used to sought patterns and connections that could be blended and others for melding to create a tentative thematic framework. The next step called ‘indexing’ enabled the association of extracts from the transcripts with themes. During ‘reviewing and refining’, thick descriptions of each theme were written, substantiated by transcript excerpts. In the final step of data analysis, namely the ‘process of critiquing’, member checking of the themes with some of the participants was done.

### Trustworthiness

Four criteria, referring to the credibility, transferability, dependability and confirmability of findings in qualitative research, were used to ensure the trustworthiness of the findings (Brink, Van der Walt & Van Rensburg 2018:158 & 159). Data were collected from 45 participants before saturation of data was obtained to ensure credibility of the findings. In addition to audio-recorded focus group interviews, field notes were used to capture data that were not reflected in the recordings and participants were involved in checking whether the identified themes portrayed the data shared during the interviews. To ensure dependability of the findings, the research methodology was thoroughly described to provide an audit trail from the inception of the research to the reporting of the findings. Confirmability of the findings was ensured by using excerpts from the interviews to substantiate the description of the categories. A dense description of the participants, the context of the study and the research methods ensures the transferability of the findings.

### Ethical considerations

Ethical clearance to conduct the study was granted by the Human Research Ethics Committee of the Faculty of Health Sciences, University of Pretoria (reference no.: 486/2022), Department of Health, Gauteng Provincial Protocol Review committee and from the designated nursing education institution management (protocol number Gp20230415). All study participants gave informed consent voluntarily to take part in the research. Their rights to freedom from harm and discomfort were respected. Confidentiality of information was ensured by using pseudonyms to replace participants’ names and surnames in the research reports.

## Results

### Demographic information of the participants

The 45 participants, who were between 22 years and 52 years old and comprised 37 females and 8 males, were allocated to different clinical facilities for work-integrated learning. They were either in the third or second year of training, and all have had previous exposure to clinical supervision during work-integrated learning (Table 1).

**TABLE 1:** Participants’ demographic information.

Gender	Age (years)	Level of study	Participants’ code
Female	30	3	G1P1
Female	29	3	G1P10
Female	31	3	G1P11
Male	31	3	G1P12
Female	23	3	G1P2
Female	29	3	G1P3
Female	29	3	G1P4
Female	26	3	G1P5
Female	26	3	G1P6
Female	26	3	G1P7
Female	20	3	G1P8
Female	22	3	G1P9
Female	22	3	G2P1
Male	45	3	G2P10
Female	23	3	G2P11
Female	31	3	G2P12
Female	23	3	G2P2
Male	23	3	G2P3
Female	30	3	G2P4
Male	34	3	G2P5
Female	24	3	G2P6
Female	20	3	G2P7
Female	37	3	G2P8
Female	29	3	G2P9
Female	37	2	G3P1
Female	23	2	G3P2
Male	40	2	G3P3
Male	24	2	G3P4
Female	28	2	G3P5
Female	24	2	G3P6
Male	23	2	G3P7
Female	52	2	G3P8
Female	25	2	G3P9
Female	24	2	G4P1
Female	26	2	G4P10
Female	26	2	G4P11
Female	31	2	G4P12
Female	22	2	G4P2
Female	26	2	G4P3
Male	29	2	G4P4
Female	43	2	G4P5
Female	25	2	G4P6
Female	31	2	G4P7
Female	52	2	G4P8
Female	24	2	G4P9

G, group; P, participant.

### Categories and sub-categories

The data were summarised into 13 categories, each one with sub-categories according to the 5-D appreciative inquiry model (Table 2). The categories refer to definitions about clinical supervision during work-integrated learning, the participants’ discovery of optimal supervision during their work-integrated learning in a variety of clinical environments, their dreams about optimal supervision, ideas about designing optimal supervision and a description of the resources and process of optimal supervision.

**TABLE 2:** Summary of categories and sub-categories.

Phases	Category	Sub-category
Definition	Being guided to apply theory into practice	Getting opportunities to practise clinical skills.
		Receiving support to perform procedures.
		Being supported to achieve learning objectives.
		Being provided with a positive clinical learning environment.
Discovery	Acknowledge existing areas of learning-conducive clinical environment	Being thankful for students’ involvement in nursing teams.
		Welcoming orientation in clinical units.
	Appreciate supportive relationships with clinical facilitators	Respecting the positive attitude of clinical facilitators.
		Valuing clinical facilitators’ willingness to teach.
		Appreciating opportunities to develop trusting relationships with clinical facilitators.
		Being grateful for the role modelling of clinical facilitators.
	Endorse opportunities for learning	Cherish opportunities to develop professionally.
		Appreciate suitable allocation to clinical learning opportunities.
Dream	Envisage a clinical learning-conducive environment	Hope for proper planning of clinical learning opportunities.
		Wish for standardised procedures in wards and clinical units.
		Dream of having sufficient time and opportunities to apply theory and master skills.
	Visualise optimal cooperation between the designated nursing education institution (NEI) and clinical facilities	Longing for cooperation that supports students.
		Recommend optimal communication to prevent students’ challenges in clinical practice.
	Wish for a favourable allocation of human and material resources to support clinical learning	Wish for enough resources to support clinical learning.
		Longing for a delegated unit in clinical facilities to support students’ learning and skills development.
Design	Improve clinical learning environment	Revision of clinical learning opportunities.
		Standardisation of nursing procedures in wards and clinical units.
		Correlation between the time allocated for and the scope of skills to be mastered.
	Support cooperation between designated NEI and clinical facilities	Enhanced cooperation between the NEI and clinical facilities to benefit students’ learning.
		Optimal communication to prevent students’ challenges in clinical practice.
	Request favourable allocation of human and material resources to support work-integrated learning	Budget allocation to meet clinical learning demands.
		A delegated unit in clinical facilities to support students’ learning and skills development.
Destiny	A clinical learning-conducive environment	Revise curriculum and clinical learning opportunities.
		Address discrepancies in procedures in wards and clinical units.
		Revisit the time allocated to apply theory and master skills.
	Optimal cooperation between NEI and clinical facilities	Improve cooperation between the NEI and clinical facilities.
		Ensure optimal communication to prevent students’ challenges in clinical practice.
	Favourable allocation of human and material resources to support clinical learning	Adjust budget to support clinical learning.
		Implement a delegated unit in clinical facilities to support students’ learning and skills development.

*Source:* Mugwari, M.J., 2024, ‘Nursing students’ appreciation of clinical supervision during work-integrated learning’, Masters dissertation, University of Pretoria

NEI, nursing education institution.

### Definition phase of appreciative inquiry

#### Being guided to apply theory into practice

The participants first described how they understood the definition of clinical supervision and work-integrated learning. They referred to clinical supervision as all the opportunities that students get to learn about, and once they are skilled, to demonstrate clinical nursing procedures under the supervision of professional nurses:

‘I could say clinical supervision is to go to the clinical facilities and to be exposed to the clinical procedures that you have been taught in theory at the college and now demonstrating them in the real-life situation.’ (Female, 52 years, level 2)

‘Clinical supervision for me is being observed while you are demonstrating the skills that you have been taught in the simulation lab and doing it by getting that demonstration in the clinical facilities.’ (Male, 23 years, level 2)

According to the participants, clinical supervision should be defined as professional nurses’ support of students during challenging circumstances. One of them verbalised it as follows:

‘In the case of going to the clinical facility, they will now show you how to do that in a clinical environment … they will guide you on how to do certain skills and how to deal with the challenges experienced in clinical areas.’ (Female, 25 years, level 2)

Nursing students require support from professional nurses to perform delegated tasks during work-integrated learning. With sufficient guidance, they are enabled to master skills and achieve learning objectives:

‘If I am doing the delegated tasks more often with the guidance and supervision of professional nurses or clinical lectures, I would be mastering the procedure and achieving my learning objectives.’ (Female, 23 years, level 3)

According to the participants, clinical supervision refers to professional nurses’ teaching of students to perform nursing procedures in learning-conducive environments:

‘When they are close to us and becoming friendly … they should be there to clarify and help us … when they involve us, whenever they are handling out any duty so that they can be able to teach us in the unit.’ (Male, 40 years, level 2)

### Discovery phase of appreciative inquiry

#### Acknowledge existing areas of learning-conducive clinical environment

In the discovery phase of the research, the participants discussed what worked best for them. They were thankful that the professional nurses involved them in nursing teams during work-integrated learning:

‘We need to collaborate and work as a team … teamwork, collaboration, communication and independence are best for me.’ (Male, 29 years, level 2)

The participants appreciated the thorough orientation to the clinical facilities, as they, after such sessions, were familiar with the available resources and the processes that were routinely used in specific wards:

‘I also feel like I work best if I am orientated in the ward and about the clinical facility’s routine.’ (Female, 26 years, level 2)

#### Appreciate supportive relationships with clinical facilitators

The participants appreciated the clinical facilitators’ and the hospital professional nurses’ positive attitude towards them. They were kind and patient with the students:

‘The operational manager is so hands-on … even the staff attitude is so positive. So that is the ward … where we meet our learning objectives.’ (Female, 23 years, level 3)

One participant alluded that she had opportunities to take part in activities in the ward under the supervision of professional nurses to enhance her knowledge and skills:

‘From the operational manager to the staff were friendly and they were willing to teach me.’ (Female, 23 years, level 3)

The participants appreciated the trusting relationships between the clinical facilitators and students that developed during supervision. It reduced their anxiety in uncertain situations and created a safe work-integrated learning environment:

‘Trusting relationships reduces the level of anxiety and the stress that we could be having regarding certain procedures and scary patients’ conditions.’ (Female, 20 years, level 3)

The clinical facilitators and operational managers displayed their role modelling expertise and inspired the participants to become skilled professional nurses:

‘She is that type of operational manager who wanted you to become a professional nurse. The way she treated the patients, she did not treat the patients like she was doing them a favour.’ (Female, 20 years, level 3)

### Endorse opportunities for learning

The participants appreciated exposure to opportunities to learn to become clinically skilled and to act in a professional manner:

‘I developed critical decision-making and was able to effectively solve real-life patients’ problems … we were resuscitating a patient … the manager who was hands-on and helpful to the sister who was on duty made it possible for me to be exposed to good clinical supervision.’ (Female, 22 years, level 3)

Another participant expressed her gratitude that she got the chance to develop communication skills:

‘I feel like it helps in developing a person’s communication skill and behaviour …’ (Female, 26 years, level 3)

Proper planning for student allocation enhances their learning and skills development, and the participants, therefore, appreciated the proper planning of their learning opportunities:

‘The organisational manager ensured that students were placed according to their learning objectives … she will ask what you are supposed to do … even the staff.’ (Female, 31 years, level 3)

### Dream phase of appreciative inquiry

#### Envisage a clinical learning-conducive environment

Most participants wished to experience proper planning of clinical learning opportunities and wished for the use of standardised procedures in all wards in all clinical facilities associated with the designated nursing education institution, as well as for having sufficient time to learn how to implement theory in practice:

‘My ideal clinical supervision would be having a clinical facilitator who will dedicate a day to one or two procedures for the whole group of the students … that day will be solely based on clinical supervision and work-integrated learning.’ (Female, 30 years, level 3)

#### Visualise optimal cooperation between the designated nursing education institution and clinical facilities

Optimal cooperation between the lecturers at the nursing education institution and the professional nurses at the clinical facilities was the wish of many of the participants. They hoped that the lecturers, clinical facilitators and the professional nurses of the clinical facilities could find ways to cooperate to the benefit of the supervision of students:

‘I dream of the ideal clinical supervision that does not punish failure or set unrealistic expectations … we are people who are not the same … they can use failure and turn it to something that strengthens the character that builds you.’ (Female, 37 years, level 3)

Advanced technology could be used to expose students repeatedly to procedure demonstrations in preparation for skills assessment:

‘I am dreaming of moving to an advanced technology kind of learning …’ (Female, 28 years, level 2)

When optimal communication between lecturers, clinical facilitators and professional nurses in the associated hospitals is maintained, students’ challenges with integrating theory into practice could be minimised:

‘With regard to the student placement for all levels, I think there should be proper consideration on the number of students that are placed in a particular ward …’ (Female, 24 years, level 2)

When standardised procedures are used in all the clinical facilities associated with the designated nursing education institution, the confusion of students who rotate to all the facilities for work-integrated learning will be prevented:

‘Like anything should be standard and clear because that way will prevent a sort of friction … if procedures are standard from both the management side and the student side … there would be no friction.’ (Female, 24 years, level 2)

Having sufficient time to apply theory in practice and to master skills may help students achieve their learning objectives during work-integrated learning. Most participants agreed that when sufficient time is allocated to work-integrated learning, it will be easy to gain competency in clinical skills:

‘My ideal clinical supervision will be allocating enough time for clinical supervision. Bearing it in mind that we are not the same, we learn differently … you will find that others will grasp the procedures very quickly and others take time …’ (Female, 37 years, level 3)

#### Wish for a favourable allocation of human and material resources to support clinical learning

Favourable allocation of human and material resources to support clinical learning emerged as the third category of the dream phase. The participants wished for enough human resources to implement quality clinical supervision:

‘My ideal clinical supervision will be that of having enough resources and equipment in both the clinical facilities and learning institutions so that we will be able to give proper quality patient care and receive quality education.’ (Female, 23 years, level 3)

‘I wish or dream of an ideal clinical supervision where we have enough professional nurses so that we can receive adequate supervision from them.’ (Female, 29 years, level 3)

The assignment of professional nurses to guide them to achieve their learning objectives would help students to gain clinical skills:

‘Delegate one professional nurse per day who will be responsible for the clinical supervision of the students … they will rotate on a daily basis so that all of them are well equipped in clinical supervision of the students.’ (Female, 29 years, level 3)

‘My ideal clinical supervision will be a place where we have someone who has been allocated for students. Someone who will guide the students … and who will be able to give the students undivided attention.’ (Male, 23 years, level 3)

### Design phase of appreciative inquiry

#### Improve clinical learning environment

In designing an optimal clinical environment, the participants wanted the opportunities for clinical learning to be revised. According to them, the assessment of students’ competency should also be revised as many shortcomings are experienced:

‘And with the clinical placement, can the institution also do proper research on whether the staff of the teaching clinics are willing to teach students.’ (Female, 30 years, level 3).

‘With the recent summative assessment … we were expected to do a procedure in 10 minutes, I think the facilitators need to go to the ward and see how it is done … and see if it is doable. If they find it working, then now can expect us to do it in 10 minutes.’ (Female, 26 years, level 3)

The participants also made suggestions on how the standardisation of nursing procedures could be done:

‘I think it also goes back to the standardisation we spoke about, so that all students get equal amounts of attention as well as time to practice their skills.’ (Female, 24 years, level 2)

In order to correlate the time allocated for skills development and the scope of the skills to be developed, the participants recommended that the structure of the nursing programme offered at the designated institution should be reviewed:

‘Time is important, allocating more time for students when going to training … needs to be done so that we get more time to practice and become competent.’ (Female, 26 years, level 3)

#### Support cooperation between designated nursing education institution and clinical facilities

The discussions revealed a need for the support of cooperation between the staff of the nursing education institution and that of the clinical facilities. Proper planning of students’ work-integrated learning is required:

‘There should be proper facility planning when allocating the students … if it is conducive for learning. This could assist to mitigate any complaints that could arise from the students regarding learning … and meeting the objectives.’ (Female, 26 years, level 3)

Another participant further stated that the NEI and clinical facilities’ staff should take part in a workshop concerning their responsibilities regarding teaching students:

‘The nursing education institution together with the clinical facilities … need to be provided with a workshop to remind them of their responsibilities and accountabilities.’ (Female, 25 years, level 2)

Annual stakeholders’ meeting comprising staff from the clinical facilities, the designated nursing education institution and student representatives where students’ challenges during clinical placement are addressed need to be arranged:

‘This group must come together and discuss the challenges that they are facing from the learners and how are they going to resolve them … his could be done each and every year to discuss about how they are going to help the learners.’ (Male, 34 years, level 3)

According to the participants, the students should be represented in the planning, execution and evaluation of clinical supervision:

‘We are probably the best people to give suggestions … how can they improve and what could they do to help us understand whatever skill is being taught. I think there needs to be more communication between the institutions and the students …’ (Female, 28 years, level 2)

#### Request favourable allocation of human and material resources to support clinical learning

The participants recommended that there should be enough resources allocated at clinical facilities for students’ skills development. They recommended that more professional nurses be appointed in the clinical facilities to help them to meet their learning objectives:

‘At the hospitals we have this shortage. If they can try to close the gaps and try to hire as many nurses as possible, so that they can see if there are enough nurses in the hospital.’ (Female, 37 years, level 2)

Another participant supported the notion by stating that enough funds should be allocated to the clinical facilities and the nursing education institution in order for them to be well-furnished with resources:

‘I think more funds should be channelled to the institutions so that we can have more resources. And then moving to like advanced ways of doing things so we need more funds to be channelled in our institutions or learning facilities.’ (Male, 40 years, level 2)

The participants suggested that there should be a team of clinical facilitators who focus only on supporting students during work-integrated learning:

‘Another thing that could be put into action is to have two independent groups of lecturers. Lecturers who are basically for work-integrated learning and lecturers who are only doing theory.’ (Female, 31 years, level 2)

### Destiny phase of appreciative inquiry

#### A clinical learning-conducive environment

The revised curriculum will imply that sufficient time is allocated to the work-integrated learning of students:

‘[*B*]alance the theory and work-integrated learning, so that we have more time of clinical exposure to gain competency.’ (Female, 26 years, level 2)

‘[*A*]llocate more time for clinical exposure so that we are competent in the skills …’ (Male, 24 years, level 2)

Quality clinical learning opportunities be made available to students to learn how to integrate theory in practice to the benefit of quality patient care:

‘Proper situational analysis where someone from the nursing education institution goes to the clinical facilities and properly assess whether it is conducive for learning and conducive for the students to meet their objectives.’ (Male, 23 years, level 3)

Students’ scope of practice to be included in the clinical learning workbooks to ensure that students are exposed to applicable learning opportunities:

‘The staff needs to know the scope of practice of a student nurse … if they can incorporate the procedures and the scope of practice of a student nurse in our workbooks and procedure manuals …’ (Female, 30 years, level 3)

Sufficient time in the structure of the learning programme allocated for students’ clinical skills development:

‘With regard to the time that we spend in the clinical facilities … the college knows that there should be certain hours for theory and certain hours for clinical placements …’ (Female, 24 years, level 2)

#### Optimal cooperation between the designated nursing education institution and clinical facilities

The staff from both the designated nursing education institution and the clinical facilities receive training on how to support students during work-integrated learning:

‘[*T*] raining to the professional nurses and the lecturers on how to support students during their time of work-integrated learning could help … to reach our destiny.’ (Female, 29 years, level 3)

Random inspections done to ensure that students receive quality supervision during work-integrated learning:

‘I also think in order to get to our destiny, I think we need to get people who do random inspections in our units … regular inspection from the higher authority should be conducted …when the boss is not there to monitor, like students get really treated unfairly …’ (Female, 20 years, level 3)

Optimal communication between lecturers and clinical facilitators to the benefit of quality student clinical supervision to be maintained:

‘Communication about where you will be allocated, should be communicated earlier if possible when receiving the master educational plan.’ (Female, 24 years, level 2)

#### Favourable allocation of human and material resources to support clinical learning

A budget to be allocated to secure human and material resources to support clinical supervision of students during work-integrated learning:

‘[*T*]o make resources available for nursing education … if we can advocate in that regard to have funds allocated to address matters of human resource and equipment resources.’ (Male, 29 years, level 2)

The institution of a delegated unit of facilitators in each clinical facility associated with the designated nursing education institution to focus primarily on the supervision of students during work-integrated learning supported:

‘Lecturers for theory and for clinical teaching …’ (Female, 25 years, level 2)

The number of professional nurses being responsible for patient care and student supervision in the clinical facilities associated with the designated nursing education institution to be increased and applicable development programmes to be implemented:

‘In the clinical facilities, they will need to employ more nurses who will be able to supervise and guide students when they are placed there. And proper staff development so that they are able to deal with the supervision of the students.’ (Male, 23 years, level 3)

## Discussion

Participants defined clinical supervision as being guided to apply theory into practice. Where opportunities to practise clinical skills are used, support from clinical facilitators is needed, and a learning-conducive environment is required. According to Al-Daken et al. (2024:4), students appreciated opportunities during work-integrated learning to observe professional nurses performing procedures and thereafter to repeat the procedures with guidance from clinical facilitators. The participants’ wishes for guidance to become clinically skilled concur with the findings of a study done by Jacob, Seif and Munyaw (2023:4), who found that clinical facilitators’ main responsibility is to enable students to gain clinical competence.

According to the participants, some professional nurses were eager to teach them. They encouraged them to use opportunities to master procedures and invited them to be part of the nursing team. The findings of a study by Thapur et al. (2023:296) emphasised that supportive clinical learning opportunities are created when students are encouraged to ask questions to get clarity about ways to perform nursing procedures and to function as members of nursing teams. A study by Saati (2023:241) revealed that students associate supportive clinical environments with positive relationships with their mentors.

A learning-conducive clinical environment is vital for students’ professional development and mental well-being. Participants revealed much appreciation of being involved in nursing teams and unit orientation they received. This statement is supported by Zhang et al. (2022:548), who emphasised that learning-conducive clinical environments, where professional nurses involve students in teamwork, stimulate students to improve their clinical knowledge and skills. On the other hand, the findings of a study by Makhaya, Lethale and Mogakwe (2023:4) indicate that professional nurses felt that a thorough orientation of students on the first day of work-integrated learning made it easy for them to mentor the students.

Learning environments that stimulate students’ development consist of well-resourced hospitals and clinics, where students are allocated for long enough periods to use appropriate learning opportunities. Insufficient time for clinical exposure has a negative impact on students’ clinical competency (Matlhaba & Nkoane 2024:20). This has been confirmed by Almekkawi and Khalil (2020:314), who revealed that insufficient time for clinical exposure of students impedes them from practising independently in a real-life environment.

The participants recommended a thorough analysis of the clinical facilities to ensure that quality learning opportunities exist before students are placed for work-integrated learning. Botlhoko, Zenani and Sehularo (2023:48) support their recommendations and emphasised that only clinical facilities with excellent learning opportunities should be used for nursing students’ work-integrated learning. Teaching programmes should continuously be revised to align students’ clinical learning objectives with the available learning opportunities in work-integrated learning (Drateru 2019:3). Discrepancy in procedures in wards and clinical units was one of the challenges, which in turn resulted in the participants’ confusion during assessments. The participants, therefore, recommended the necessity to standardised nursing procedures and that the procedures should be comprehensively described in clinical manuals. These recommendations corroborate with research findings of Mbakaya et al. (2020:435), who stated that expert clinical facilitators should be appointed and procedure manuals standardised to prevent student confusion. Fadana and Vember (2021:5) suggested that clinical facilitators should be trained on how to demonstrate the procedures to prevent students’ confusion. The clinical facilitators should, according to Ti-Enkawol Nachinab and Armstrong (2024:6), ensure that the professional nurses of the clinical facilities associated with nursing education institution implement the standardised procedures during patient care.

### Strengths and limitations

Although the study was conducted at a designated nursing institution, the recommendations can be implemented at other institutions in similar contexts. A large sample of participants were interviewed, but the male students were outnumbered by the female students. Only eight male students took part. There was, unfortunately, not an equal distribution of male and female students.

### Recommendations

Students are concerned about their progress to meet clinical learning objectives during work-integrated learning, and they should, therefore, be supported by all professional nurses involved in their training. Lecturers, clinical facilitators and the professional nurses in the healthcare settings, who supervise their work-integrated learning, should demonstrate to them standardised nursing procedures. Learning-conducive clinical environments are required, and the students should be allocated to the environment long enough to acquire competency. Close cooperation between the staff of the nursing education institution and the associated healthcare facilities to enable the collaborative development of clinical learning opportunities is non-negotiable.

## Conclusion

Nursing students require clinical supervision during work-integrated learning and appreciate guidance regarding theory-practice integration. Their professional growth is enhanced when they observe professional nurses delivering quality patient care. Students appreciate opportunities to practise nursing procedures under supervision. Close cooperation between the management of the nursing education institution and the associated healthcare services is recommended to ensure that quality learning opportunities are created in work-integrated learning. Without acknowledging and responding appropriately to students’ feedback about challenges in work-integrated learning, valuable opportunities to support their competency development are lost. The authors call on the management of nursing education institutions to diligently plan, execute and evaluate students’ work-integrated learning to the benefit of the profession and the patients that the profession serves.
